# Listener effort measures clinically meaningful change of dysarthria in amyotrophic lateral sclerosis

**DOI:** 10.1093/braincomms/fcaf232

**Published:** 2025-06-12

**Authors:** Indu Navar Bingham, Raquel Norel, Esteban G Roitberg, Julián Peller, Marcos A Trevisan, Carla Agurto, Michele Merler, Diego E Shalom, Felipe Aguirre, Iair Embon, Alan Taitz, Donna Harris, Amy Wright, Katie Seaver, Stacey Sullivan, Jordan R Green, Lyle W Ostrow, Ernest Fraenkel, James D Berry

**Affiliations:** Peter Cohen Foundation dba Everything ALS, Seattle, WA 98112, USA; IBM Research Center, Yorktown Heights, NY 10598, USA; Peter Cohen Foundation dba Everything ALS, Seattle, WA 98112, USA; Escuela de Ciencia y Tecnología, Universidad Nacional de San Martín, Buenos Aires B1650, Argentina; Peter Cohen Foundation dba Everything ALS, Seattle, WA 98112, USA; Laboratorio de Neurociencias Cognitivas Computacionales, Humai, Buenos Aires C1425, Argentina; Universidad de Buenos Aires, Facultad de Ciencias Exactas y Naturales, Departamento de Física—CONICET—Universidad de Buenos Aires, Instituto de Física Interdisciplinaria y Aplicada (INFINA), Buenos Aires C1428, Argentina; IBM Research Center, Yorktown Heights, NY 10598, USA; IBM Research Center, Yorktown Heights, NY 10598, USA; Universidad de Buenos Aires, Facultad de Ciencias Exactas y Naturales, Departamento de Física—CONICET—Universidad de Buenos Aires, Instituto de Física Interdisciplinaria y Aplicada (INFINA), Buenos Aires C1428, Argentina; Peter Cohen Foundation dba Everything ALS, Seattle, WA 98112, USA; Peter Cohen Foundation dba Everything ALS, Seattle, WA 98112, USA; Peter Cohen Foundation dba Everything ALS, Seattle, WA 98112, USA; Peter Cohen Foundation dba Everything ALS, Seattle, WA 98112, USA; Department of Neurology, Lewis Katz School of Medicine at Temple University, Philadelphia, PA 19140, USA; Peter Cohen Foundation dba Everything ALS, Seattle, WA 98112, USA; Peter Cohen Foundation dba Everything ALS, Seattle, WA 98112, USA; MGH Institute of Health Professions, Charlestown Navy Yard, Boston, MA 02129, USA; Peter Cohen Foundation dba Everything ALS, Seattle, WA 98112, USA; Sean M. Healey and AMG Center for ALS, Massachusetts General Hospital & Harvard Medical School, Boston, MA 02114, USA; MGH Institute of Health Professions, Charlestown Navy Yard, Boston, MA 02129, USA; Department of Neurology, Lewis Katz School of Medicine at Temple University, Philadelphia, PA 19140, USA; Department of Biological Engineering, Massachusetts Institute of Technology, Cambridge, MA 02139, USA; Sean M. Healey and AMG Center for ALS, Massachusetts General Hospital & Harvard Medical School, Boston, MA 02114, USA

**Keywords:** amyotrophic lateral sclerosis, ALS, dysarthria, quantitative motor speech, listener effort

## Abstract

Amyotrophic lateral sclerosis (ALS) is a neurodegenerative motor neuron disease that can cause progressive bulbar dysfunction and dysarthria, resulting in reduced quality of life. Quantitative motor speech analysis can identify features of dysarthria that worsen with ALS progression but are not, inherently, clinically meaningful. Listener effort (LE) is a clinician-rated feature describing how much effort the listener needs to exert to understand the dysarthric speaker. This study investigated whether LE could act as a clinically meaningful measure of ALS dysarthria that could be used as an outcome measure in clinical trials. The Everything ALS Speech Study obtained longitudinal clinical information and speech recordings from 292 participants. In a subset of 125 participants, we measured speaking rate and three speech–language pathologists (SLPs) with expertise in ALS rated LE. We also built and tested a LE prediction algorithm to predict the SLPs’ rating of LE. In addition, all speech recordings and associated clinical data are now being made available to ALS researchers via the Everything ALS portal. LE intra- and inter-rater reliability was very high (ICC 0.94–0.95). LE correlated with other measures of dysarthria at baseline and changed over time in participants with ALS (slope 0.77 pts/month, SE = 0.15, *P* < 0.001) but not controls (slope 0.005 pts/month, SE = 0.02, *P* = 0.807). The slope of LE progression was faster in people with bulbar onset than non-bulbar onset ALS (1.66 points/month versus 0.42 pts/month; *P* < 0.001) but was similar in all participants who had bulbar dysfunction at baseline, regardless of ALS site of onset (1.52 pts/month for bulbar onset versus 0.98 pts/month for non-bulbar onset with current bulbar involvement; *P* = 0.36). The LE prediction model predicted the true LE, with an average *R*^2^ of 0.83 ± 0.07. Dysarthria is associated with decreased quality of life in people with ALS. Quantitative measures of dysarthria in ALS could be useful as ALS clinical trial outcome measures, providing insight into the progression of bulbar symptoms. Speaking rate quantifies progression but is variable across speaking stimuli, emotional states and contextual factors. LE is more inherently clinically meaningful, can be measured reliably by SLPs, changes quantitatively over time and is highly reproducible, thus may be useful as a clinical outcome assessment for ALS clinical trials. Furthermore, a LE prediction model is effective at predicting LE scores and should be validated on an external dataset.

## Teaser

Amyotrophic lateral sclerosis (ALS) causes complex progressive dysarthria. Listener effort (LE) is an endpoint that quantifies the severity of dysarthria in other diseases. The authors show that LE measured by speech pathologists or by a machine learning model can quantify ALS progression and could be used as a trial endpoint.

## Graphical Abstract

**Graphical Abstract:**
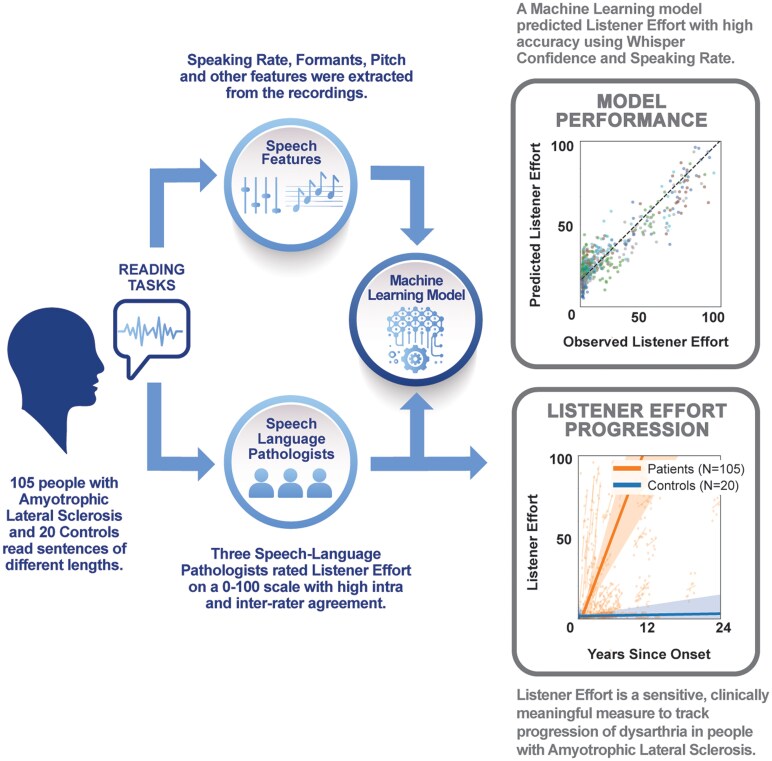

## Introduction

Amyotrophic lateral sclerosis (ALS) causes progressive weakness of muscles under voluntary control throughout the body. It begins in the bulbar region in approximately 25–30% of people.^1^ Progressive bulbar dysfunction and dysarthria eventually develops in 80–95% of people living with ALS.^2^ The ALS Functional Rating Scale—Revised (ALSFRS-R) is traditionally used to quantify disease progression,^3^ and the ALSFRS-R Self-Entry (i.e. self-reported; ALSFRS-RSE) correlates highly with the ALSFRS-R and progresses at a similar rate.^4,5^ Thus, ALSFRS-RSE is frequently used in remote ALS studies. Despite its frequent use, the ALSFRS-R (and ALSFRS-RSE) is a blunt instrument for testing dysarthria, since only one question (Q1) asks about speech intelligibility, which is rated 0–4, and only three questions (Q1–3) focus on bulbar function (ALSFRS-R bulbar subdomain).

Quantitative motor speech (QMS) analysis describes standard analyses aimed at quantifying characteristics or features of speech such as rate, pause or articulation. QMS is conducive to remote studies because it can be implemented using speech recordings obtained in the home environment on personal electronic devices (smartphones, tablets or computers), allowing frequent, simple data collection. In ALS, QMS has focused on speaking rate (SR; words/min), articulation rate (AR; syllables/s) and speech pause analysis (SPA) to identify speech abnormalities, which, in some instances, can be detected even before people with ALS (PALS) or their speech–language pathologists (SLPs) are aware of them.^4,6-10^ However, each of these features may be insufficient to quantify ALS progression over time.

A clinically meaningful outcome measure quantifies a change in an outcome that has real importance for the way a person feels, functions or survives. Dysarthria significantly impacts communication and quality of life. ALS causes complex progressive dysarthria. This dysarthria can occur due to dysfunction in any one or more of the speech subsystems (articulatory, resonatory, phonatory and respiratory).^11,12^ And these speech subsystems can decline at different rates, while numerous compensatory mechanisms can further complicate patterns of dysarthria. Thus, while a reliable quantitative measure of overall dysarthria severity in ALS will require complex modelling to incorporate features from different speech subsystems, such a measure will be clinically meaningful.^11,13^ Specific measures of individual speech subsystems may also quantify clinically meaningful progression of dysarthria but must be correlated to other clinically meaningful outcome measures, such as patient-reported outcome measures, to imbue them with clinical meaning.^9^

SLP ratings of percent intelligibility,^14^ for example, have demonstrated modest reliability at quantifying the severity of dysarthria in ALS^15^ and correlate with PALS self-reports of dysarthria and resulting distress.^16,17^ These measures have even been used to estimate the time to loss of intelligibility in people living with ALS—approximately 32 months for people with bulbar onset ALS.^14^ Listener effort (LE) is a perceptual rating of the amount of work necessary for a listener to understand a speaker with disordered speech.^18^ It has been used extensively as a patient-reported outcome measure of hearing impairment.^19,20^ In the context of ALS, LE is applied differently, as an assessment made by a healthy listener to quantify the negative impact of dysarthria;^18^ and, it has proven to be one of the most robust overall measures of dysarthria.^15^

In this study, we aimed to conduct a fully remote observational study characterizing speech in ALS. We focused on speech recordings to characterize SR and AR. Because AR, SR and other QMS features only characterize individual facets of dysarthria, and ALS is characterized by complex degeneration of multiple speech subsystems, we sought to identify a more clinically interpretable overall measure of progressive dysarthria in ALS. SLPs with ALS expertise rated LE to evaluate its performance as a digital clinical outcome assessment (COA) in ALS. Finally, we developed a machine learning model to predict LE, the listener effort prediction model (LEPM).

The repository of recorded speech and de-identified clinical data from this study is now available to ALS researchers to advance speech research (see Data Availability).

## Materials and methods

### Speech study

The Everything ALS Speech Study was designed to gather speech recordings and patient-reported outcomes (PROs) from people living with ALS to create a data and speech recording portal to share with the research community.

Enrolment in the study began in January 2021, and the study was designed to be fully remote to enable diverse and rapid enrolment. Participants were recruited through social media outreach, email outreach through ALS advocacy organizations, and the Everything ALS community webinars. Speech recordings included in this report were acquired between 3 January 2021 and 31 May 2023.

#### Tasks

The session was guided by a virtual dialogue agent through four different structured speech tasks in the following order: (i) reading a 99-word passage known as the Bamboo Passage,^21^ (ii) Picture Description of an image randomly chosen from a pool of 23 unique images,^22^ (iii) Sentence Reading of 5-, 7-, 9-, 11-, 13- and 15-word sentences randomly chosen from a pool containing at least 15 sentences per category^23^ and (iv) performing a diadochokinetic rate task (DDK) which consists of a quick repetition of the syllables ‘puh-tuh-kuh’ until the participant run out of breath. Only the recordings from the Sentence Reading task were used in this study.

### Ethics oversight

All aspects of the design and conduct of the Everything ALS Speech Study were approved by the Western IRB. Every participant is presented with a digital written informed consent through a study portal and provides written documentation of informed consent prior to undergoing any study procedure, in accordance with the Declaration of Helsinki.

All participants consented to inclusion of their study data (for example, coded identifiers, demographics, ALS history and outcome measures and speech samples) in a large speech database that can be accessed by researchers under appropriate data use agreements.

### Compliance, completeness and study dashboards

Non-interventional ALS studies consistently demonstrate approximately 50% loss-to-follow-up at 6 months.^24,25^ Over the course of this study, the Everything ALS team developed participant retention strategies focused on platform improvements and frequent contact between the study team and participants. Participants received email reminders about their required sessions each week with the embedded secure link to record with one click. A programmable fit-for-purpose data dashboard was developed to allow (i) the Everything ALS study team to monitor enrolment, engagement, compliance and data quality and (ii) participants to track their engagement and data. Participants could decide if they wanted to see their past ALSFRS-RSE score, with it displayed on a separate screen. Speech features from the reading passage, including speaking rate (wpm), DDK (syllables per second) and loudness (dB, uncorrected for head-to-mic distance) were presented on an adjustable time graph to measure trend over time on the dashboard. Key statistics of the three features of the last session (SR, DDK and loudness), the last 30-day average and the all-time average were presented in a tabular form.

As a result of ongoing data quality and compliance review, university student volunteers were added as ambassadors to the Everything ALS study team to increase team outreach to participants, answer technical questions and encourage higher compliance. All student ambassadors undergo onboarding and compliance training, including for the remote delivery of the Edinburgh Cognitive Assessment Scale (ECAS) using a computer-based, examiner delivered form of the scale.^26^

### Data acquisition

The Everything ALS Speech Study uses a web-based platform from Modality.ai to present speech tasks to participants, an automated assistant to guide participants through the tasks, and video and audio to capture the results of speech tasks.

Speech and video recordings were obtained from participants as frequently as weekly with no restrictions on the number of recordings, although participants could record new sessions even if they missed prior sessions.

### ALS Functional Rating Scale—Revised Self-Entry

The self-entry form of the ALSFRS-R (ALSFRS-RSE) was presented to participants as a part of the Modality.ai data collection, along with speech recordings. The ALSFRS-R Self-Entry (ALSFRS-RSE) is very highly correlated with the ALSFRS-R at baseline (though ALSFRS-RSE is generally 1–3 points higher) and has a similar slope of decline.^4,5,27,28^ In this study, it is used to anchor speech analyses to the functional status of participants.

The Rasch Overall Disability Scale (ROADS)^29^ was delivered at alternating sessions until October 2022, when it was removed to reduce the burden of study visits. ROADS data are not presented in this manuscript but are available in the shared data.

### Data analysis

Demographics and compliance rates were tabulated using descriptive statistics (Table 1). QMS analysis has become relatively common in ALS research.^4,6,8,10,11^  ^14^ Here, we use the Whisper automatic transcriber to compute SR. To improve accuracy, the first and last words were excluded due to border effects that produce unreliable timestamps. The rate was then calculated by dividing the number of remaining words by the time span between their timestamps. AR follows a similar methodology but removes the pauses and divides syllables instead. To calculate the number of syllables, we followed a simple heuristic that counts syllables in a word by identifying vowel clusters. It increases the count for a vowel following a non-vowel, adjusts for silent ‘e’ endings and accounts for ‘le’ endings. It ensures at least one syllable is counted, even for edge cases. Correlations between motor speech features and other clinical characteristics, such as ALSFRS-RSE were performed using Pearson R.

**Table 1:** Demographics and ALS disease characteristics of speech study and LE sub-study

	Overall speech study			LE sub-study		
	All	PALS	Ctrl	All	PALS	Ctrl
*N*	292	136	156	125	105	20
Age (median, range)	65 (30–85)	66 (32–83)	63 (30–85)	67 (31–85)	67 (39–83)	67 (31–85)
Sex (*n*)						
Female	173	67	106	60	50	10
Male	119	69	50	65	55	10
Ethnicity (*n*)						
Non-Hispanic	273	128	145	116	98	18
Hispanic	14	5	9	6	5	1
Unknown	5	3	2	3	2	1
Race (*n*)						
White	265	124	141	112	94	18
Asian	13	5	8	5	5	0
American Indian or Alaska Native	6	3	3	4	3	1
Black or African American	4	2	2	1	1	0
No data	4	2	2	3	2	1
Baseline ALSFRS-R (mean, std)	42.3 (7.7)	36.1 (7.2)	47.8 (0.9)	38.1 (7.7)	36.2 (7.0)	47.9 (0.3)
Diagnostic Delay [time between the onset of weakness and diagnosis (years)]		1.4 (1.4)			1.4 (1.5)	
Time since ALS symptom onset at baseline (years)		3.9 (3.7)			4.1 (3.8)	
Reported ALS inheritance (*n*)						
Familial		48			40	
Sporadic		88			65	
Onset location (*n*)						
Bulbar		34			23	
Non-bulbar		101			81	
Unknown		1			1	

### Speech recording quality assurance

Every session was screened for quality by the Modality.ai platform^30^ Sessions were excluded if users experienced technical difficulties such as poor internet connection, session restarts or non-starts, device issues (e.g. system errors) or premature session termination. Sessions were further screened for completeness. If a participant did not complete all the tasks in a session, the session was labelled incomplete. Additionally, sessions were discarded if they had either corrupted or non-existent WAV files.

### LE sub-study

#### Pilot studies

Two pilot studies were conducted before the full LE sub-study.

*First pilot.* The purpose of the first pilot was to identify clinically meaningful measures of motor speech for further study and to select the speech tasks on which to evaluate those measures. In this first pilot, we investigated recordings of three reading tasks: the Bamboo Passage, the Picture Description and Sentence Reading (11, 13 and 15 words in length, randomly selected from a pool of 19, 16 and 18 sentences, respectively). Each task was rated using 11 outcome measures: LE, Overall Dysarthria Severity, Slow Speaking Rate, Voice Strain, Consistency, Reduced Intelligibility, Articulatory Imprecision, Dysphonia Severity, Hypernasality, Reduced Breath Support and Reduced Prosody. To constrain variability, we employed clinicians trained in dysarthria and ALS (SLPs: D.H., A.W., K.S., and S.S. S.S. only participated in the first pilot, later replaced by A.W.).

Based on its performance in this first pilot study, the task selected for further study was Sentence Reading, and the outcome measure selected was LE, marked on a 0–100 scale of increasing effort. The rationale for this decision was straightforward: the sentences were short and easy to score, allowing for multiple ratings from the same session; the sentences were selected out of pools to reduce learning effects for both readers and raters.

For the selected task, each SLP scored a total of 30 sentences (3 sentences from 10 participants). The inter-rater reliability in this first stage was 0.75, 0.97 and 0.71 for each pair of raters, as measured by the intraclass correlation coefficient (ICC), with an overall inter-rater reliability of 0.79. After the first pilot, the SLPs met to discuss and unify evaluation criteria, focusing especially on the ratings with higher disagreements.

*Second pilot.* The purpose of the second pilot study was to test and evaluate the user interface (UI) developed for the full sub-study. Thirty new recordings of the 11-, 13- and 15-word sentences were scored by the SLPs, this time only on LE. The inter-rater reliability for this second stage was 0.91, 0.90 and 0.88 for each pair of raters, with a global inter-rater reliability of 0.90, showing that the training and first pilot helped improve the agreement between raters.

Additionally, a multi-talker babble noise feature (3 dB SNR) was built into the platform under the hypothesis that it could help differentiate between low and medium intelligibility scores. When toggled on, this feature introduced a layer of unintelligible babble to distract listeners. Multi-talker babble places a cognitive load on the listener, making it harder to rely on contextual cues and forcing greater attention to degraded speech signals. This makes even mild articulatory imprecision or timing disruptions more evident. Because the multi-talker babble noise was found not to impact intelligibility scores, it was not carried forward into the full sub-study.

### Full LE sub-study

There were 125 participants in the LE sub-study (105 ALS and 20 controls matched by age and sex; see Table 1 for demographics), all of whom had at least 2 months of speech recording data. Each session consisted of three recordings from the 11-, 13- and 15-word sentences uttered by a PALS, resulting in a complete session comprising nine ratings (three SLPs rating three sentences each).

**Figure 1:**
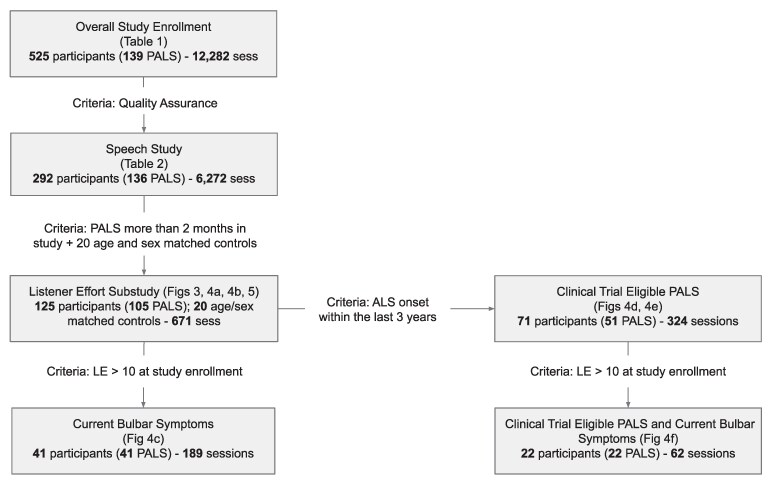
**Cohort diagram.** Flow of participants from enrolment in the overall study, through quality assurance, inclusion in the overall speech study, inclusion in the listener effort (LE) sub-study and creation of cohorts within the LE sub-study. Criteria for inclusion in sub-studies and cohorts are noted. Cohorts described in specific tables and figures throughout the study are noted.

Each SLP rated 2549 recordings (7647 total ratings). This included 20% of recordings that were presented twice to calculate intra-rater reliability (Fig. 1 and Table 2). Thus, the initial dataset for the longitudinal LE analysis included a unique set of 2124 speech recordings rated by the SLPs, corresponding to 708 sessions.

**Table 2:** LE speech recording sessions collected, quality controlled and analysed

	Patients	Controls	Total
Participants	105	20 (10 M, 10 F)	125
Sessions before QC	584	124	708
Sessions after QC	552	119	671
Average sessions per participant after QC	5.26	5.95	5.37
Timespan in study (days)	348	386	354
Unique speech samples before quality control	1749	375	2124
Total speech samples included after quality control	1605	348	1953
20% repetitions	325	78	403
Total speech samples	1930	426	2356

We implemented a set of filtering steps as a quality assurance (QA) process to ensure the reliable extraction of speech features, which are fundamental to our analysis, particularly Speaking Rate (computed based on the Whisper transcripts). The following three steps represent the minimal necessary filtering to maintain data quality and integrity:

1. *SLPs notes*. The notes taken by the SLPs on the audio files contain valuable information about the PALS in unstructured natural language. These notes often include keywords related to speech characteristics (e.g. accent, dysarthria) or audio quality (e.g. noise, low volume). There were 125 recordings marked with the keywords ‘noise’, ‘volume’, ‘quality’, ‘recording’, ‘sample’, ‘error’, ‘nothing’ or ‘no speech’ that we excluded to avoid errors in automatic transcriptions using Whisper. These recordings were rated solely as a prerequisite of the platform to proceed to the next recording, rather than as an actual assessment of the recording. This prevents quantifying the effect of this step on the LE distribution. This left us with 1999 usable recordings (out of the original 2124) from 692 sessions with at least one rated sentence.
2. *Automatic transcriptions.* Automatic transcriptions may produce hallucinations—instances where the model fabricates content or misinterprets audio, introducing non-existent information that affects the calculation of Speaking Rate and Whisper Confidence. To ensure that calculated features corresponded to the presented sentences, we included only recordings matching at least 20% with the original text. This left us with 1970 recordings without hallucinations (out of the 1999 remaining after the first filter) and 688 sessions with at least one recording. The distribution of LE scores in the discarded recordings differed significantly from the full dataset (Mann–Whitney U = 486940, *P* < 0.0001). Although this filter tended to exclude recordings with high dysarthria, with mean LE scores tending towards higher dysarthria values (M = 84.8, SD = 24.4), 82% of the cases of severe dysarthria (LE > 70) were retained. This step excluded 18% of recordings rated with severe dysarthria (LE > 70).
3. *Minimum number of rated recordings.* The third filter ensures comparable LE averages across sessions. Standard sessions consist of nine ratings (three sentences rated by three SLPs); we excluded those sessions that ended up with only one recording (three ratings, one from each SLP), due to the previous two filters. This filter excluded only 17 sessions, leaving us with 1953 recordings (out of the 1970 remaining after the second filter), corresponding to 671 sessions (out of the 688 sessions). The distribution of LE scores in the discarded recordings was not significantly different from the full dataset (Mann–Whitney U = 163701, *P* < 0.234).

Raters used a web-based rating platform with secure login and volume normalization that presented speech recordings one at a time (Supplementary Fig. 4). All raters were provided with Sennheiser 280 Pro headphones and underwent hearing screening in the 60–70 dB SPL amplitude range. The system repeated 20% of all recordings randomly to assess intra-rater reliability. Recordings were presented in a random order. At each session, a ‘warm-up’ listening task was presented to prepare raters. A digital Visual Analogue Scale (VAS), guided with a mouse or finger, was used for the rating. The scale was 0 (easily understandable) to 100 (unintelligible even with full effort).

Of note, during the second pilot, the virtual visual analog slider used to select the LE score by each rater was initially positioned at 50 and did not require the rater to move the slider to record a score, which resulted in raters inadvertently scoring recordings a 50. For the full LE sub-study, the system was updated to require the slider to be moved prior to recording a score. One rater misunderstood, incorrectly assuming that the slider could not be moved back to exactly 50, thus, did not rate any recordings as exactly 50.

### LE statistical analysis

Intra- and inter-rater reliability for LE was analysed using interclass correlation (ICC). Intra-rater reliability was calculated using 20% of the audio recordings that were rated twice. Following existing guidelines, inter-rater reliability was calculated using all the audios (for those rated twice, we kept the first rating).^31^ ICCs were interpreted as follows: <0.5 poor reliability, 0.5–0.75 moderate reliability, 0.75–0.9 good reliability and >0.90 excellent reliability.

The final LE for each participant in a session was computed as follows: Each session consists of three sentences. Three SLPs listened to each of these sentences, providing a total of nine ratings per session. The LE score for each participant’s session was determined by averaging these nine ratings.

Contingency tables were created to investigate the relative sensitivity to change of the LE metric, ALSFRS-RSE Q1, ALSFRS-RSE bulbar subdomain, ALSFRS-RSE total score and Speaking Rate (Table 3). Change over time was dichotomized into either ‘Progression’ (the participant showed more decline than the maximum seen in the control population) or ‘No Progression’ (the participant did not show more decline than the max in the control population). The number of participants showing Progression was evaluated for each measure, with the measures identifying more participants with progression being the most sensitive to change.

**Table 3:** Comparison of the number of participants (*n*) with ‘Progression’ or ‘No Progression’ based on longitudinal changes in the ALSFRS-R Q1, bulbar subdomain, total score and speaking rate compared with LE

		ALSFRS-R Q1		ALSFRS-R bulbar subdomain		ALSFRS-R total score		Speaking rate	
		NP	P	NP	P	NP	P	NP	P
LE	NP	10	3	11	2	2	11	13	0
	P	25	67	30	62	8	84	80	12

NP, no progression; P, progression*.

*Progression is defined as a score that declined more than the maximum change in the control population over the period of observation.

A change in LE was evaluated using both unbiased and traditional modelling approaches. First, an unbiased machine learning method called mixture of Gaussian processes (MoGP) was used to identify groups of participants with similar progression rates on LE. MoGP identifies clusters of participants with similar rates and patterns of decline for a given outcome measure. It has been described for clustering participants based on the ALSFRS-R decline.^32^

To evaluate the overall progression rate of LE, a linear mixed model with ‘participant’ as a random effect was applied. This model was used to determine whether the LE slope differs from zero. The study populations included (i) controls, (ii) PALS and (iii) PALS filtering those who had normal LE levels at the outset of the study. Further analysis within the PALS group was conducted using a linear mixed model, again with ‘participant’ as a random effect, to explore differences based on group assignment. Group comparisons included (i) participants with bulbar onset ALS versus non-bulbar onset ALS to determine if the slopes of decline differ based on the site of onset and (ii) participants with bulbar onset ALS versus non-bulbar onset ALS excluding all participants with normal LE at the outset of the study, to determine whether, once bulbar symptoms have begun, the progression rate varies depending upon whether onset was bulbar or non-bulbar.

The longitudinal coefficient of variation (CoV) was calculated to enable a comparison of the residual error compared with the amount of change in outcome measures, including SR, ALSFRS-R Q1 and LE. CoV was also calculated for ALSFRS-RSE, as a point of reference. CoV could be calculated in several ways. Because the CoV in this case was being used to provide some insight into the performance of these variables as outcome measures in a clinical trial, CoV was calculated based on the linear mixed model slope estimate for each variable, such that CoV = Standard error of slope/Mean slope. As such, the CoV allows comparison of the degree of residual variability standardized by the slope of change. Variables with CoV closer to 0 have less variability per unit change of the slope.

### LE prediction model

Given the robust performance of the LE, a statistical model was built to predict LE. The model, called LEPM, was built using a Lasso regression model fed with acoustical features. The Least Absolute Shrinkage and Selection Operator (Lasso) is a type of linear regression that incorporates regularization through an L1 penalty.^33^ This penalty term is proportional to the absolute value of the coefficients, leading to some coefficients being shrunk to zero. This model allows both predicting and identifying relevant features by excluding those with zero coefficients.

### Acoustic features

A set of standard features was chosen from a wide range of commonly reported markers in literature for tracking bulbar decline. These features include mean and standard deviation of pitch, mean and standard deviation of formants 1 and 2, standard deviation of the sound envelope, harmonic-to-noise ratio, shimmer, jitter and cepstral peak prominence (CPP). All these features were computed using the Parselmouth library.^34^ We also included two features of the speech system: speaking rate and Whisper confidence.

### Model evaluation

In our study, SLPs rated 708 sessions, from which we extracted features comprehensively. Following the QA process, 671 sessions were retained for further analysis. A Lasso regression model was trained and evaluated using nested cross-validation with five outer folds and five inner folds within each outer fold. Each outer fold included 25 unique participants (PALS and controls). Uniformity of the target distribution across folds was confirmed via a Kolmogorov–Smirnov test between each outer fold and the remaining combined folds; this procedure was similarly applied within the inner folds, which comprised 20 unique participants each. Cross-validation within each inner fold determined the optimal level of regularization for the models. Subsequently, the best-performing model from each outer fold was retrained using data from the other four outer folds and tested on the remaining fold.

## Results

### Study enrolment and demographics

In total, 525 participants entered the study and recorded at least 1 session, 457 recorded at least 2, 412 recorded 3 or more (range of recording numbers: 1–144). After matching all participants’ recording and clinical data and passing data through quality control, the dataset included 292 participants (136 with ALS and 156 controls) with a total of 6272 speech recording sessions for a total of 56 462 individual speech recording tasks (Fig. 1).

Of the 292 participants, 40.8% were male and 59.2% were female, and the median age was 65 (range 30–85). In this study, 91% of PALS were white, far more than reported in the US National ALS Registry of 71%^35^ but similar to other ALS clinical studies^24,36^ (Table 1). The participants were enrolled from 15 states, though 27% did not provide geographic information (Supplementary Fig. 1).

### Motor speech characteristics at baseline and longitudinally

In this cohort, the average baseline SR for PALS was 145.9 words/min (SE 3.8) and for controls was 186.1 words/min (SE 1.6; *P* < 0.001), and the average baseline AR for PALS was 3.339 syllables/s (SE 0.085) and for controls was 4.269 syllables/s (SE 0.036; *P* < 0.001).

Longitudinal analysis of AR for PALS shows a slope of −0.0035 units/month (SE 0.0013; *P* = 0.010) and for controls 0.0125 units/month (SE 0.0010; *P* = 0.23). Longitudinal analysis of SR for PALS shows a slope of −0.203 units/month (SE 0.079; *P* = 0.011) and for controls shows a slope of 0.34 units/month (SE 0.45; *P* = 0.45) (Supplementary Fig. 2A and D).

People with bulbar onset ALS had a slower speech at baseline than non-bulbar onset [bulbar onset 117.6 words/min (SE 10.6); non-bulbar 170.7 words/min (SE 5.1); *P* < 0.001] (Supplementary Fig. 2, panels B and E). However, those experiencing bulbar symptoms at study entry had similar speaking rates at baseline and progression of slowing over time regardless of site of ALS onset [non-bulbar with bulbar symptoms at study entry 117.6 words/min (SE 10.3), bulbar onset 110.8 words/min (SE 7.6); *P* = 0.59] (Supplementary Fig. 2C and F).

### LE sub-study

#### Intra- and inter-rater reliability of LE is high

**Figure 2:**
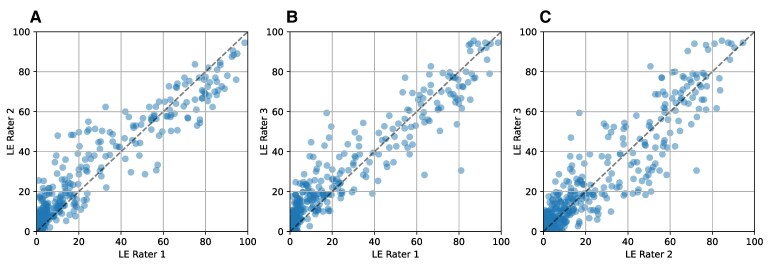
**Listener effort (LE) inter-rater reliability.** Each point represents a session (*n* = 671 sessions). Pairwise inter-rater reliability was excellent for all three pairs of raters: (**A**) LE Rater 1 and 2 intraclass correlation coefficient (ICC) was 0.95 (*P* < 0.001); (**B**) LE Rater 1 and 3 ICC was 0.95 (*P* < 0.001). (**C**) Inter-rater reliability was very good for the third pair: LE Rater 2 and 3 ICC was 0.94 (*P* < 0.001).

Intra-rater reliability, as measured by interclass correlation (ICC), was excellent for two raters (0.92 and 0.91) and very good for one rater (0.89). The ICC did not differ significantly for each sentence’s task (11, 13 and 15 words). Pairwise inter-rater reliability ICC was excellent for two of the three rater pairs (0.92, 0.91) and very good for the third pair (0.88) (Fig. 2).

#### LE correlates with other features of bulbar function and declines as self-reported speech declines

**Figure 3:**
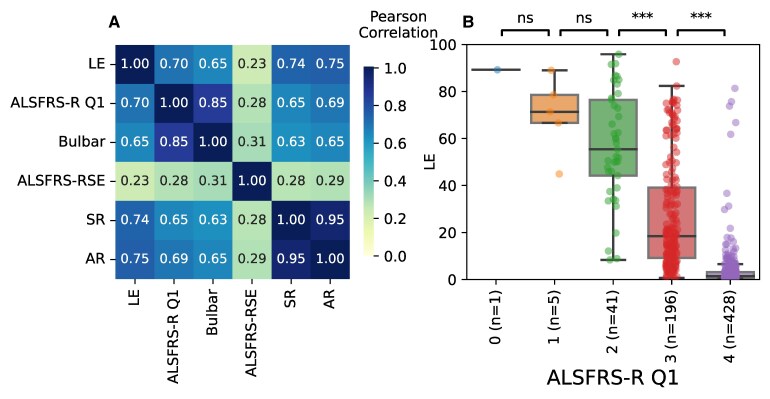
**Listener effort (LE) versus the ALS functional rating scale-revised self-entry (ALSFRS-RSE) and acoustic features.** (**A**) Correlation matrix was computed over the population of people with ALS (PALS) (*n* = 552 sessions). Correlations are expressed in absolute values. Speaking rate (words/min; SR) and articulation rate (syllables/s; AR) are remarkably highly correlated (Pearson *R* = 0.95, *P* < 0.001), indicating that they measure the same aspect of speech. There is a good correlation between LE and AR (*R* = −0.75, *P* < 0.001), SR (*R* = −0.74, *P* < 0.001), ALSFRS-R Question 1 (*R* = −0.70, *P* < 0.001) and ALSFRS-RSE bulbar subdomain (*R* = −0.65, *P* < 0.001). This indicates that while LE measures similar concepts to these measures, it also contributes non-overlapping information. As expected, because ALSFRS-RSE covers many more domains than just speech, it shows a low to moderate correlation with AR, SR and LE. (**B**) LE of each level of ALSFRS-R Q1, for the entire population. Each point corresponds to a session (*n* = 671 sessions). In participants with lower self-reported speech function on the speech question (Q1) of the ALSFRS-RSE, LE increases (lower ALSFRS-RSE scores, and higher LE, denote lower speech function). Because Q1 of the ALSFRS-RSE only has five categorical answers, each category contains a wide spread of LE scores. These differences are significant comparing the categorical answers 2–3 (Mann–Whitney test, U = 6524, *P* ≤ 0.01) and 3–4 (U = 78216, *P* < 0.01), but not 0–1 (U = 5, *P* = 0.33) and 1–2 (U = 136, *P* = 0.25). This may be because of the low numbers of participants in the lowest categories of ALSFRS-RSE Q1.

There were moderate-high to high correlations between LE and other measures of ALS and dysarthria severity, suggesting that they measure related but not completely overlapping content. Mean LE correlated well with the Speech question (Q1) on the ALSFRS-RSE (Pearson *R* = −0.70, *P* < 0.001) and the bulbar subdomain (Q1–3) (*R* = −0.65, *P* < 0.001). LE also correlated well with SR (*R* = −0.74, *P* < 0.001) and AR (*R* = −0.75, *P* < 0.001) (Fig. 3A and B). SR and AR did not correlate as well with ALSFRS-RSE Q1 (*R* = −0.65, *P* < 0.001 and *R* = −0.69, *P* < 0.001, respectively) or the bulbar subdomain (*R* = −0.63, *P* < 0.001 and *R* = −0.65, *P* < 0.001, respectively) (Fig. 3A).

Both AR and SR measure speech speed and contain essentially the same information (*R* = 0.95, *P* < 0.001). Therefore, we retained only SR in subsequent analyses.

#### Unbiased clustering based on LE slope of decline defines distinct subgroups

We conducted an unbiased clustering of LE slopes using MoGP on the 46 PALS with onset <3 years prior to study entry and at least two audio sessions. The analysis revealed two distinct patterns of progression: non-progressors (Cluster A, *n* = 22, slope = 0.05 pts/month, SD = 0.40; Cluster B, *n* = 8, slope = 0.03, SD = 12.34) and progressors (Cluster C, *n* = 8, slope = 3.26 pts/month, SD = 9.18; Cluster D, *n* = 11, slope = 3.76 pts/month; SD = 23.44) (Supplementary Fig. 3). The unbiased identification of these clusters suggests that while the pace and pattern of dysarthria progression are variable, there are patterns to the progression.

#### PALS with bulbar onset have faster progression than those with non-bulbar onset ALS

**Figure 4:**
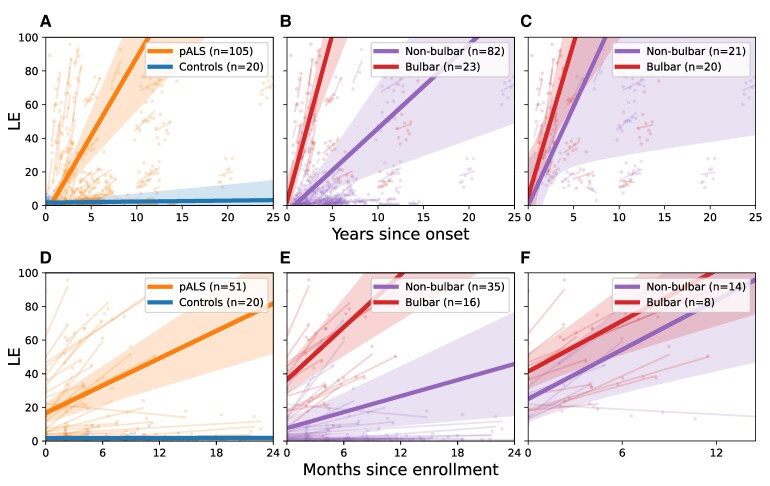
**Progression of listener effort (LE) in people with ALS (PALS) and controls.** We analysed ALS progression using linear mixed models (LMM) in different cohorts. In panels **A–C**, we plotted LE data since onset from all participants, and in panels **D–F**, we plotted LE data since enrolment from participants with onset of ALS within 3 years of study initiation. In panels **A** and **D**, we compare PALS and controls. (**A**) The slope of LE was 0.77 points/month for PALS (SE = 0.15, *P* < 0.001) and 0.005 points/month for controls (SE = 0.02, *P* = 0.807). (**D**) For PALS with onset <3 years before enrolment, the slope was 2.71 points/month (SE = 0.53, *P* < 0.001) and 0.005 points/month for controls (SE = 0.02, *P* = 0.807). In panels **B** and **E**, we compare PALS with bulbar and non-bulbar onset. (**B**) PALS with bulbar onset had a LE progression rate of 1.66 points/month, while non-bulbar onset PALS had a smaller slope of 0.42 points/month (*P* < 0.001). (**E**) For PALS with onset <3 years before enrolment, those with bulbar onset had a slope of 5.21 points/month, and those with non-bulbar onset had a smaller slope of 1.58 points/month (*P* < 0.001). In panels **C** and **F**, we compare PALS with bulbar and non-bulbar onset, excluding PALS with LE scores in the normal range (0–10) at the time of enrolment. This partition focuses the analysis on PALS with current bulbar symptoms. (**C**) LE slope for PALS with bulbar onset was 1.52 points/month and 0.98 points/month for non-bulbar onset, showing no statistical differences (*P* = 0.364). (**F**) For PALS with onset <3 years before enrolment, the slope was 4.89 points/month for bulbar onset, and 5.02 for non-bulbar onset, showing no statistical differences (*P* = 0.946). This suggests that once participants have developed bulbar symptoms, LE tends to progress at a similar rate, whether the disease began in the bulbar region or not.

The slope of decline of LE was 0.76 pts/month (SE = 0.15, *P* < 0.001) for PALS and 0.005 pts/month (SE = 0.020, *P* = 0.807) for controls (Fig. 4A). PALS with bulbar onset ALS (*n* = 23) had a faster LE progression rate (1.66 pts/month) than non-bulbar onset (*n* = 82) whose LE progression rate was 0.42 pts/month (*P* < 0.001; Fig. 4B). Patterns were similar among PALS with onset <3 years prior to study entry, a population more resembling that of an ALS clinical trial population (Fig. 4D and E).

#### Normal LE at baseline in PALS predicted participants likely to have slow progression

The LE slope for PALS with LE 0–10 at baseline (*n* = 64) was only 0.03 pts/month, not significantly different than zero (*P* = 0.13). When we included only PALS with onset within the last 3 years, mimicking a trial population, the progression rate for those with LE 0–10 at baseline (*n* = 29) was 0.87 pts/month, which was significantly different than zero (*P* = 0.027), but still slower than PALS with higher baseline LE (*n* = 22) whose slope was 5.2 pts/month (*P* < 0.001).

#### Regardless of site of onset, once bulbar symptoms begin, LE progresses at similar rates

LE progression rates in PALS with bulbar onset and those with limb onset whose disease had progressed to involve bulbar function by the time of study enrolment were compared. Twenty PALS had bulbar onset ALS and 21 PALS had limb onset and bulbar involvement at baseline, defined as LE > 10. The LE decline was not different between these two groups (1.52 pts/month for bulbar onset, 0.98 pts/month for limb onset with current bulbar involvement; *P* = 0.36; Fig. 4C and F). The pattern was similar for PALS with symptom onset <3 years prior to study entry. SR slopes were also not significantly different between these groups.

##### Sample size calculation for clinical trial

Using the population of 51 PALS with onset less than 3 years before enrolment (Fig. 4D), we calculated the required sample size assuming 90% power, a 0.05 significance level and a 30% mean difference between treatment groups. Based on an observed slope of 2.71 pts/month and a standard deviation of 3.42, the estimated sample size is 187 participants in each group. This calculation follows the method described in Rutkove *et al*.^28^ Although our data collection approach differs from the high-frequency sampling in Rutkove *et al*., this estimation provides meaningful insights into the feasibility of using LE as an endpoint in ALS trials.

#### LE is more sensitive to the progression of bulbar symptoms over time than ALSFRS-RSE

In the LE sub-study, 92 of the 105 PALS showed ALS progression (increase over time) in their LE score. By contrast, only 70 showed progression on Q1 of the ALSFRS-RSE, 64 on the bulbar subdomain, and 12 on SR, indicating that LE is more sensitive at detecting change over time than ALSFRS-RSE Q1, bulbar subdomain and SR (Table 3). Ninety-five PALS showed progression in ALSFRS-RSE total, a smaller increase over LE than we hypothesized.

The longitudinal coefficient of variation (CoV) of SR, ALSFRS-RSE Q1 and LE were calculated on the same set of sentences. CoV was 0.40 for SR, 0.29 for ALSFRS-RSE Q1 and 0.20 for LE. For reference, the CoV for ALSFRS-RSE total score was 0.14.

#### Model prediction of LE

##### LEPM predicts LE with high accuracy

**Figure 5:**
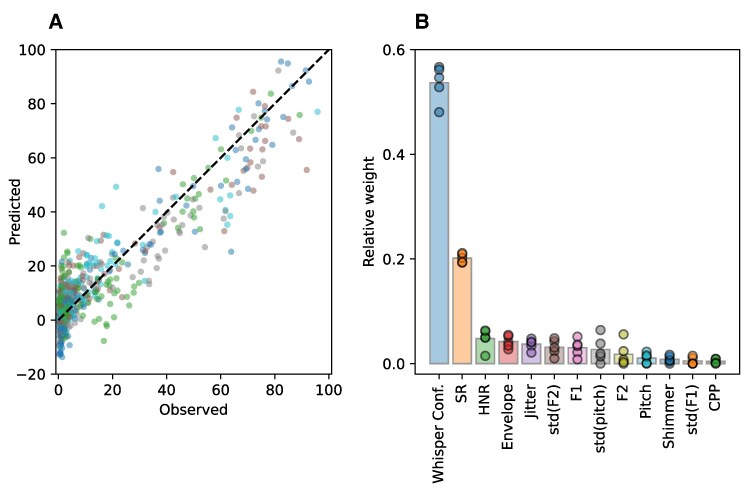
**Listener effort prediction model (LEPM).** (**A**) A Lasso regression model was trained and evaluated using nested cross-validation. The predicted LE output is plotted on the *y*-axis against the LE as assessed by SLPs on the *x*-axis. Each data point represents a single session (*n* = 671), where each outer fold is shown in a different colour. The model showed a robust performance: the RMSE averaged across the five outer folds was 8.56 ± 0.60, and the average *R*^2^ was 0.83 ± 0.07 (*P* < 0.001). (**B**) The relative weight of each feature averaged across the outer folds. Data points represent the relative weight of a feature in one of the five outer folds. Most of the predictive power of the LEPM comes from Speaking Rate and Whisper Confidence.

The model showed a robust performance: the root mean square error (RMSE), averaged across the five outer folds was 8.56 ± 0.60, and the average *R*^2^ was 0.83 ± 0.07 (Fig. 5A). Notably, most of the predictive power of the LEPM comes from two key features: speaking rate and whisper confidence (Fig. 5B).

#### The Everything ALS Speech Recording and Clinical Data Portal makes data and speech recordings available to researchers

All the speech recordings and associated de-identified metadata are available to researchers via the Everything ALS Data Portal. The portal is hosted on Amazon Web Services in a HIPAA-compliant secure environment. Participants are identified in the study by a NeuroStamp, a coded identifier specific to the Everything Austen ALS Speech Study that was derived from the NeuroGUID (global unique identifier). NeuroGUID is a coded identifier used to aggregate records from the same participants without the need for re-identification. NeuroStamp preserves the ability to aggregate data without exposing the NeuroGUID, adding an extra layer of de-identification.

Self-reported clinical data, including overall health information, ALS disease history and routine outcome measures, including ALSFRS-RSE and speech recordings (uncompressed .wav format) are hosted in the data portal. Speech recordings and data can be requested online (see Data Availability).

## Discussion

This study has demonstrated that speech can be collected from a large cohort of participants with ALS over time using a remote study design with good participant retention and compliance, thanks to the design and participant engagement elements of the study. Our results confirm prior findings that SR changes over time with ALS progression. However, SR captures only one aspect of the complex and variable dysarthria of ALS.

LE, a clinician-reported COA, may provide a more holistic measure of effective communication in conditions with progressive and multifactorial impacts on speech. In this study, LE was more sensitive to change than the ALSFRS-RSE Q1 or the bulbar subdomain and other QMS features. LE is practical to apply to ALS clinical trials as the assessment can be done on recordings by traditional cell phones, efficiently and reproducibly scored by SLPs using a simple online platform. While not tested in this study, we expect that LE could be implemented for global ALS trials, since SLPs who speak the same languages as trial participants are part of multidisciplinary care teams in other countries.

SLP ratings of LE showed very high intra- and inter-rater reliability. LE correlated with the bulbar subdomain of the ALSFRS-RSE and with other motor speech features, including speaking and articulation rates, but also added new information to the analysis. LE also provided interesting new insights into bulbar ALS progression. Most notably, when we excluded patients who had not yet developed bulbar symptoms at trial enrolment, the slope of LE decline was the same for participants who had bulbar or limb onset ALS. The same phenomenon was true for SR. This suggests that once bulbar symptoms start, regardless of the initial site of ALS symptom onset, dysarthria progresses at a similar rate.

In ALS clinical trials, where the goal of treatment is to slow or halt progression, outcome measures must record a decline over the period of observation. Those participants who do not show a decline over the trial duration are non-informative and reduce trial power. Thus, measures more sensitive to change over time provide higher statistical power. LE detected a change in bulbar function with more sensitivity than the ALSFRS-RSE bulbar subdomain, suggesting that it might add statistical power to clinical trial analyses relative to a bulbar subdomain analysis.

The employment cost for three SLPs to produce LE ratings is relatively modest within the budget framework of a clinical trial, in which speech data is being collected. The platform we utilize for generating these ratings is highly cost-efficient. At the same time, once trained and validated in external datasets, the LEPM provides a high-quality alternative to SLP ratings at extremely low cost, reducing expenses and enabling the calculation of LE ratings almost instantaneously, improving the practicality and efficiency of the measure for clinical trials.

Not only is LE a quantitative endpoint of bulbar function, it is also an inherently clinically meaningful endpoint since it reliably measures dysarthria, a critical factor affecting how patients feel, function and survive. The effect of dysarthria on communication and, in turn, quality of life, is well accepted in clinical settings. The EFNS-ALS guidelines^37^ suggest assessment of communication and treatment with communication support systems. People living with ALS worry about losing the ability to communicate.^38,39^ Decreased speech function on the ALSFRS-R question 1 (speech) is associated with poorer quality of life (QoL) on the ALS-Specific QoL Questionnaire (ALSSQoL).^40^ Furthermore, augmentative communication devices stabilize or improve both the quality of life and mood in people with dysarthria due to ALS.^41,42^ Similar impacts of progressive dysarthria on quality of life have been demonstrated in Parkinson’s disease^43^ and other neurological disorders.^44^

Finally, our team generated a machine learning model to predict LE, as rated by SLPs that was remarkably effective at predicting LE scores. Future studies to further validate the LEPM with external data sets are planned.

The Everything ALS Speech Data Repository is the largest ALS speech dataset available for broad use by ALS researchers and will facilitate method improvement and clinical trial modelling for the entire field (see Data Availability). It will allow broader access to annotated speech recordings to hasten speech research, and benefit ALS trial design and drug development.

## Supplementary Material

fcaf232_Supplementary_Data

## Data Availability

All the speech recordings and associated de-identified metadata are available to researchers via the Everything ALS Data Portal. Self-reported clinical data, including overall health information, ALS disease history and routine outcome measures, including ALSFRS-RSE and speech recordings (uncompressed .wav format) are hosted in the data portal. Speech recordings and data can be requested via an online form at https://www.EverythingALS.org/available-data. The repository https://github.com/EverythingALS/LEClinicallyMeaningfulDysarthriaALS contains the data used to generate the figures and tables in this work.
