# Supplementary material for: Listener effort measures clinically meaningful change of dysarthria in amyotrophic lateral sclerosis
Source: Brain Commun. 2025 Jun 12;7(4):fcaf232. doi: 10.1093/braincomms/fcaf232 (PMC12301878; doi:10.1093/braincomms/fcaf232)
Supplement: fcaf232_Supplementary_Data [file fcaf232_supplementary_data.pdf]

Listener effort measures clinically meaningful change of dysarthria in amyotrophic lateral sclerosis

Supplementary Material

	Geography (All N=291)
N	State
30	New Jersey
22	California
17	New York
14	Pennsylvania
12	Illinois
10	Massachusetts
9	Texas
8	Washington, Maryland
7	Michigan, North Carolina, Tennessee
6	Indiana, Missouri
5	Oregon
4	Colorado, Minnesota
3	Florida, Arkansas, Virginia
2	Georgia, West Virginia, South Carolina, Arizona, Ohio, Washington, D.C., Connecticut, Oklahoma
1	Alaska, Vermont, Kentucky, New Hampshire, Maine, Kansas, South Dakota, Alberta (Canada), Louisiana, Manitoba (Canada), Nevada, Alabama
78	No data

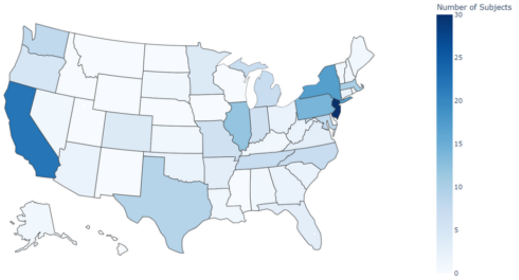

Example: there are three participants from Florida, three from Arkansas, and three from Virginia

Supplementary Figure 1: **Geographic distribution of the Speech Study**

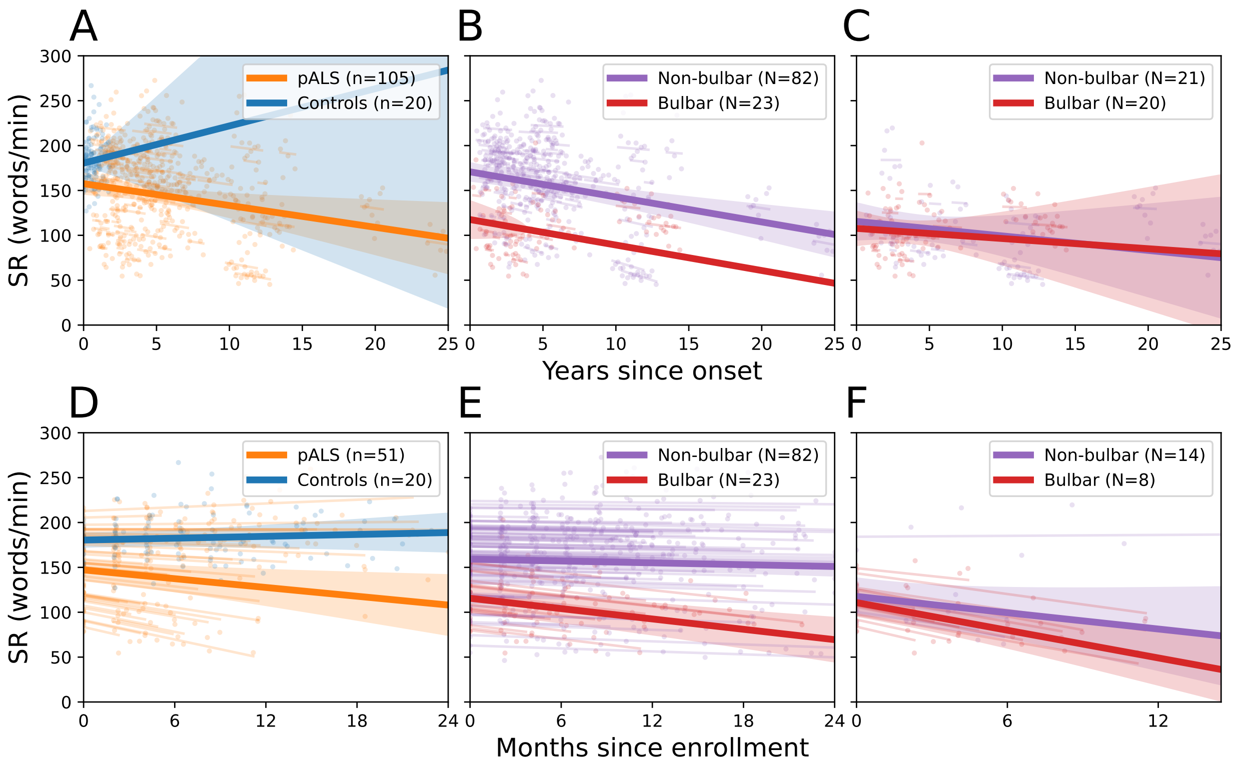

Supplementary Figure 2: **Progression of Speaking Rate in People with ALS (PALS).** We analyzed ALS progression using linear mixed models (LMM) in different cohorts. In panels (A-C) we plot speaking rate (SR) data since ALS onset from all participants, and in panels (D-F) we plot SR data since study enrollment for participants with onset of ALS within 3 years of study initiation. In panels (A) and (D) we compare PALS and controls. The slope of decline of SR is higher for PALS than controls in both all participants and those with onset ¡3yrs prior to enrollment. The positive slope and the dispersion of the regression for controls can be partly explained because the data from the controls are concentrated in a very short time range compared to the patients. In panels (B) and (E) we compare PALS with bulbar and non-bulbar onset. (B) PALS with bulbar onset surprisingly show a similar slope of decline but a slight offset in y-intercept compared to those with non-bulbar onset when considering data since ALS onset, and (E) the expected faster slope of decline when considering only those with onset ¡3yrs prior to study enrollment. In panels (C) and (F) we compare PALS with bulbar and non-bulbar onset, excluding PALS with listener effort (LE) scores in the normal range (0-10) at the time of enrollment. This partition focuses the analysis on PALS with current bulbar symptoms. In this analysis, SR slopes for PALS with bulbar and non-bulbar onset show no statistical differences. In aggregate, this analysis still suggests that once participants have developed bulbar symptoms, SR tends to progress at a similar rate, whether the disease began in the bulbar region or not.

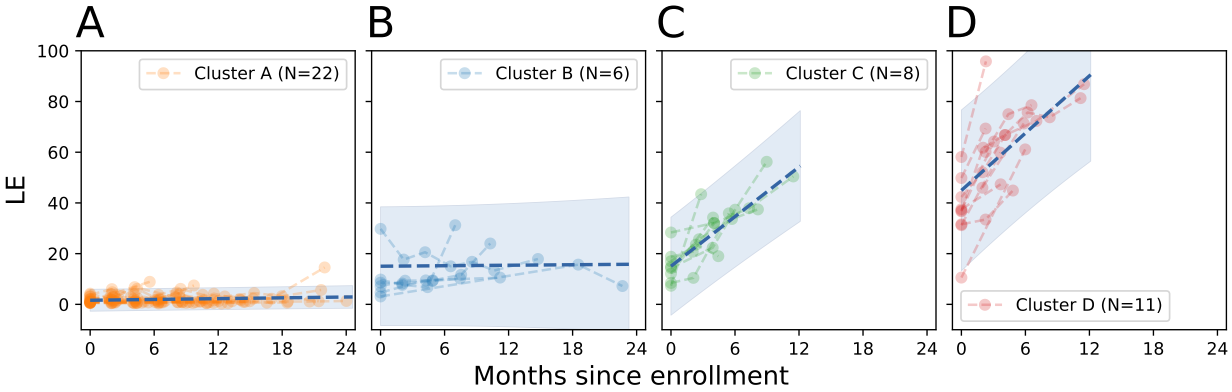

Supplementary Figure 3: **Mixture of Gaussian Processes (MoGP) Clusters for LE.** Using the MoGP to cluster change in LE, four main clusters are created, roughly characterizing non-progressors (A and B), those with slow change (C), and those with faster change (D). This suggests that even unbiased modeling approaches can identify clinically meaningful subpopulations within the larger group.

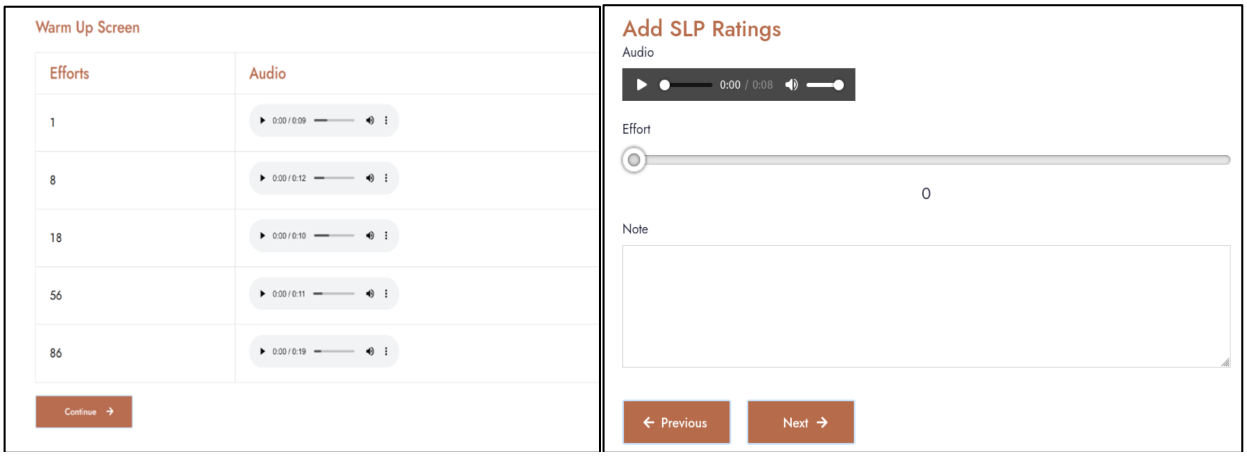

Supplementary Figure 4: **Speech and Language Pathologist (SLP) Interface for Listening to Recordings and Rating LE.**
